# The complete chloroplast genome of *Prunus japonica* thunb.(Rosaceae), an ornamental and medicinal plant

**DOI:** 10.1080/23802359.2020.1848477

**Published:** 2021-01-13

**Authors:** Jing Mu, Yuyang Zhao, Yali He, Jiahui Sun, Qingjun Yuan

**Affiliations:** National Resource Center for Chinese Meteria Medica, China Academy of Chinese Medical Sciences, Beijing, China

**Keywords:** *: Prunus japonica*, chloroplast genome, phylogenetic analysis

## Abstract

*Prunus japonica* is an ornamental and medicinal plant that is widely cultivated. The complete chloroplast genome of *P. japonica* was sequenced using Illumina Hiseq X Ten platform. The chloroplast genome was 158,080 bp in length, containing two short inverted repeat (IRa and IRb) regions of 26,385 bp, which was separated by a large single copy (LSC) region of 86,270 bp and a small single copy (SSC) region of 19,040 bp. The GC content of the whole chloroplast genome was 36.8%. The chloroplast DNA of *P. japonica* comprised 112 distinct genes, including 78 protein-coding genes, 4 ribosomal RNA genes and 30 transfer RNA genes. Phylogenetic analysis indicated that all species of *Prunus* formed a monophyletic group, *P. japonica* was closely related to *P. hulimis*.

*Prunus japonica* Thunb., also called Korean bush cherry, is a shrub species in the genus *Prunus* of family Rosaceae, which is widely cultivated for ornamental and medicinal use. Its native range extends from Northeast and East China to Korea (Shi et al. 2013). The seed kernel can be used as medicine, which is named Yu Li Renin China, that has significant antihypertensive effect.

Chloroplast genomes are important sources for phylogenetic analyses, genetic diversity evaluation, and plant molecular identification (Dong et al. 2017, 2018; Sun et al. 2020). In this study, we determined the complete chloroplast genome (cpDNA) sequence of *P. japonica* based on the next-generation sequencing method. The annotated cpDNA has been deposited into GenBank with the accession number MT991008.

Samples of *P. japonica* were collected from Quanjiao county, Anhui province, China (32°5′38″N, 118°16′7″E). Voucher specimen was stored at the herbarium of Institute of Chinese Materia Medica (CMMI), China Academy of Chinese Medical Sciences with the specimen voucher number is 341124LY0988. Total genomic DNA was isolated from fresh leaves using a DNeasy Plant Mini Kit (QIAGEN, Valencia, California, USA) according to the manufacturer’s instructions. And the sequencing library was constructed and quantified following the methods introduced by Dong et al. (Dong et al. 2017). Paired-end (150 bp) sequencing was performed by Novogene Bioinformatics Technology Co.Ltd. (Beijing, China), using the Illumina Hiseq X-Ten platform. Next-generation sequencing QC toolkit was used for quality control and to filter the low quality reads. Contigs were assembled from the high quality paired-end reads by using the SPAdes 3.6.1 program (Kmer = 95) (Bankevich et al. 2012). The chloroplast genome contigs were selected by the Blast program (Altschul et al. 1990), taken the cp genome of *P. mongolica*(GenBank: KY073235) as the reference. The selected contigs were assembled using Sequencher 4.10. Gene annotation of *P. japonica* was performed using DOGMA annotation (Wyman et al. 2004). Where necessary, the positions of start and stop codons and boundaries between introns and exons were manually corrected.

The circular cpDNA of *P. japonica* was 158,080 bp in length, containing two short inverted repeat (IRa and IRb) regions of 26,385 bp, which was separated by a large single copy (LSC) region of 86,270 bp and a small single copy (SSC) region of 19,040 bp. The GC content of the whole chloroplast genome was 36.8%. The cpDNA of *C. formosana* comprised 112 distinct genes, including 78 protein-coding genes, 4 ribosomal RNA genes and 30 transfer RNA genes. In these genes, 19 were duplicated in the IR regions and 19 genes contained one or two introns.17 harbored a single intron, and two (*ycf3*、*clpP*) contained double introns.

A phylogenetic tree was constructed to confirm the location of *P. japonica* based on Seventy-eight common coding genes which extracted from Twenty-five chloroplast genome sequences, including two outgroup samples and 22 samples of Rosaceae from the GenBank. All the sequences were aligned using MAFFT (Katoh et al. 2019) and ambiguous alignment regions were trimmed by Gblocks (Castresana 2000). We conducted a maximum likelihood (ML) analysis using IQ-tree with 1000 bootstrap replicates (Nguyen et al. 2015; Zhang et al. 2020). The phylogenetic analysis revealed that samples of *Prunus* were strongly supported as a monophyletic tree (Figure 1) and *P. japonica* was closely related to *P. hulimis*. The complete chloroplast genome reported in this study will be a valuable resource for future studies on genetic diversity, taxonomy, and phylogeny of family Rosaceae.

**Figure 1.**
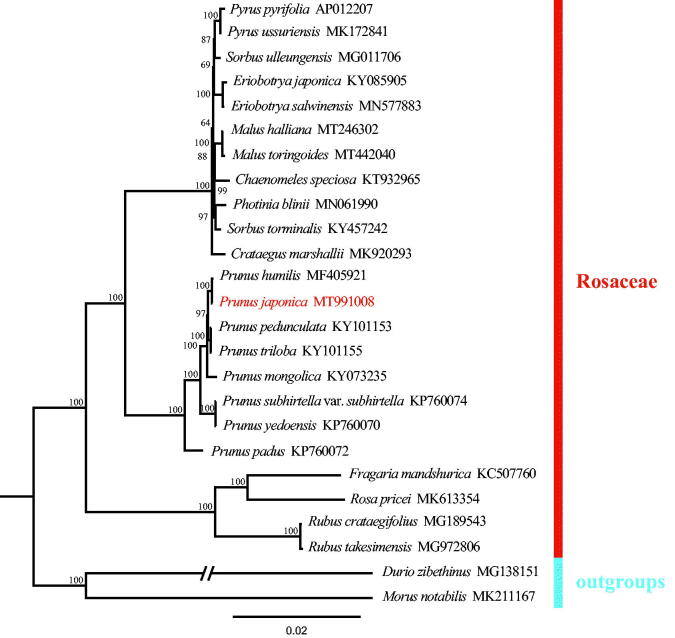
Phylogenetic tree reconstruction of 25 taxa using maximum likelihood (ML) methods based on 78 protein-coding genes in the chloroplast genome sequences. ML bootstrap support value presented at each node.

## Data Availability

The data that support the findings of this study are openly available in GenBank of NCBI https://www.ncbi.nlm.nih.gov/, reference number MT991008, raw data accession ID: SAMN16132682, BioProject ID: PRJNA660005.
